# Comparative Evaluation of Classic Treatment and Novel Intrauterine Infusion for Retained Placenta in Buffaloes: Impacts on Reproductive Performance and Economic Losses

**DOI:** 10.3390/ani15050711

**Published:** 2025-03-01

**Authors:** Stefan Coman, Daniel Ionut Berean, Raluca Cimpean, Simona Ciupe, Ioan Coman, Liviu Marian Bogdan

**Affiliations:** 1 Department of Reproduction, Faculty of Veterinary Medicine, University of Agricultural Sciences and Veterinary Medicine Cluj-Napoca, Calea Manastur 3-5, 400372 Cluj-Napoca, Romania; coman.stefan25@yahoo.com (S.C.); simona.ciupe@usamvcluj.ro (S.C.); liviu.bogdan@usamvcluj.ro (L.M.B.); 2 Department of Animal Breeding and Food Safety, Faculty of Veterinary Medicine, University of Agricultural Sciences and Veterinary Medicine Cluj-Napoca, Calea Manastur 3-5, 400372 Cluj-Napoca, Romania; calina-raluca.cimpean@usamvcluj.ro; 3 DSVSA Brasov, 500483 Brașov, Romania; ioan_coman63@yahoo.com

**Keywords:** retained placenta, buffalo reproduction, intrauterine therapy, uterine involution, microbial flora, histological evaluation, reproductive performance, economic impact

## Abstract

Retained placenta is a significant reproductive disorder in buffaloes, leading to delayed uterine involution, increased postpartum infections, and economic losses due to extended calving intervals and reduced fertility. This study aimed to compare the effectiveness of an intrauterine puerperal treatment containing vitamins, kaolin, boric acid, and neomycin with the classic method of manual extraction followed by oxytetracycline pessaries. A total of 86 buffaloes from six commercial farms in Romania were randomly assigned to either the puerperal treatment group or the classic treatment group. Uterine involution was assessed through ultrasonography and transrectal palpation, while microbial flora and histological evaluations were performed to determine uterine health. The results indicated that the puerperal-treated group exhibited significantly faster uterine recovery, a lower prevalence of pathogenic bacteria, and improved reproductive parameters, including shorter service period and calving interval. Histopathological findings showed reduced inflammation and enhanced endometrial regeneration in the treatment group. Economic analysis revealed that the improved fertility outcomes led to cost reductions associated with extended non-productive days, repeated inseminations, and milk yield losses. These findings support the use of intrauterine therapy as a superior alternative to conventional management, contributing to enhanced reproductive efficiency and economic sustainability in buffalo farming.

## Simple Summary

This study compared an intrauterine infusion (Puerperal) treatment containing vitamins, kaolin, boric acid, and neomycin with the classic method for managing retained placenta in buffaloes. A total of 86 buffaloes from six Romanian farms were divided into two groups. The puerperal-treated group showed faster uterine involution, lower pathogenic bacterial presence, and improved reproductive performance, including shorter service periods and calving intervals. Histological analysis revealed reduced inflammation and better tissue regeneration. Economic evaluation indicated lower costs due to improved fertility and reduced milk yield losses. The findings suggest that intrauterine therapy is a more effective and economically beneficial alternative to classic retained placenta management in buffaloes.

## 1. Introduction

Retained placenta (RP) in bovines is a significant postpartum complication that can lead to severe reproductive issues if not managed promptly. Traditional treatments have included manual removal and administration of oxytocin or antibiotics. Recent advancements and studies have introduced alternative methods that show promise in treating RP in buffaloes [1].

Retained placenta (RP) in buffaloes leads to significant economic losses and adversely affects reproductive performance. While specific data on buffaloes are limited, insights can be drawn from studies on dairy cows. For instance, a study assessing the economic impact of RP in dairy cows found that financial losses per incidence averaged $350.4 in primiparous cows and $481.2 in multiparous cows. The primary contributors to these losses were reductions in milk production (38.5% of the total loss) and decreased fertility (28.5% of the total loss) [2].

In buffaloes, RP has been associated with increased risks of uterine infections, such as endometritis and toxic puerperal metritis, which can prolong the calving-to-conception interval and reduce overall fertility. Additionally, RP can lead to decreased milk production and increased treatment costs, further impacting the economic viability of buffalo farming operations [3,4].

Intrauterine infusion of oxytetracycline has been utilized to treat RP in buffaloes. A study indicated that this method could effectively manage RP, leading to improved reproductive parameters in treated animals [4]. While primarily studied in cows, injection of bacterial collagenase into umbilical arteries has shown effectiveness in releasing retained fetal membranes. This procedure is considered simple, safe, and cost-effective. Although specific studies on buffaloes are limited, this method presents a potential area for further research and application in buffalo RP management [5]. In certain regions, buffalo owners have employed herbal mixtures to address RP. For example, a combination of jaggery (250 g) and carom seeds (10–15 g) has been administered to affected animals. While such practices are based on traditional knowledge, scientific validation is limited, and their effectiveness may vary [6]. The administration of oxytocin is a common practice to stimulate uterine contractions and facilitate the expulsion of the placenta. A case study reported the successful treatment of RP in a Murrah buffalo using intramuscular injections of 40 IU of oxytocin, repeated after four hours, resulting in the expulsion of the placenta within six hours post-treatment [7]. Prevention of RP is crucial and can be achieved through proper management practices, including adequate nutrition, mineral supplementation, and overall herd health management. Ensuring adequate blood glucose and calcium levels has been associated with a reduced incidence of RP.

While traditional methods remain in use, exploring and validating new treatment approaches such as intrauterine infusion and hormonal therapy can enhance the management of retained placenta in buffaloes. Further research is necessary to adapt treatments such as umbilical cord injections of collagenase, proven effective in cows, for use in buffaloes. Implementing preventive management practices is equally important to reduce the occurrence of RP. The aim of our study was to evaluate and compare the efficacy of an innovative intrauterine infusion treatment designed to dissolve the retained placenta and neutralize the unwanted effects of this pathology with the classic treatment approach for retained placenta in buffaloes, which typically involves manual removal of the placenta and intrauterine administration of antibiotics. This new treatment utilizes a product containing a combination of vitamins, kaolin, boric acid, and neomycin. By analyzing parameters such as postpartum uterine recovery and subsequent reproductive performance, our study aims to assess whether this novel intrauterine product offers improved therapeutic outcomes, enhanced reproductive efficiency, and better animal welfare compared to the traditional method.

## 2. Materials and Methods

### 2.1. Animals and Experimental Design

This study was conducted on 86 buffaloes diagnosed with retained placenta from six farms in Romania. These farms had herds ranging from 18 to 138 milking females. The buffaloes included in the study ranged from their first to sixth parturition. The animals were randomly divided into two equal groups of 43 buffaloes each: the puerperal group (P group) and the control group (C group). The P group received an intrauterine infusion of a puerperal treatment product, while the C group was treated using the classic method involving manual extraction of the placenta followed by the intrauterine administration of three pessaries containing oxytetracycline (Pasteur Romania).

### 2.2. Treatment Protocols

For the treatment group, the intrauterine puerperal treatment solution was formulated using a combination of essential vitamins, antimicrobial agents, and absorbent compounds. The final composition per 100 mL included 250,000 IU of vitamin A, 50,000 IU of vitamin D3, and 50 mg of vitamin E, derived from a vitamin mixture (Bio-vita ad3e, Biotur, Alexandria, Romania) administered at a dosage of 2.5 mL per 100 mL of solution. Additionally, boric acid (300 mg) was incorporated for its antiseptic properties, along with 100 mg (0.1 g) of neomycin as an antibiotic agent. The mixture also contained 20% kaolin (20 g per 100 mL) to facilitate absorption and toxin binding, while distilled water was added to complete the volume to 100 mL. The puerperal product was administered intrauterinely at a standardized dose of 200 mL on the second day postpartum. Administration was performed by cervical fixation and introduction of a catheter attached to a 100 mL syringe through the vulvar commissure after post-cervical sectioning of the placenta.

For the control group, manual extraction of the placenta was performed using aseptic techniques, followed by the intrauterine insertion of three (1.5 g) oxytetracycline pessaries (Oxyvet pessaries, Biotur, Alexandria, Romania) to reduce the risk of postpartum uterine infections.

### 2.3. Uterine Involution Monitoring

Uterine involution was monitored on days 7, 14, 21, and 28 postpartum using ultrasonography (Easy Scan, Maravet, Baia Mare, Romania) equipped with a linear probe (3–7.5 MHz) and transrectal palpation. Uterine size was measured using the Grunert scoring method [8]. The scores were as follows:

- Score 1: uterus retracted, uterine horn ≤ 2 cm.
- Score 2: uterus retracted, uterine horn of 3–5 cm.
- Score 3: uterus retracted, uterine horn of 6–8 cm.
- Score 4: margins of the uterus delimited by hand, uterine horn of 9–20 cm.
- Score 5: part of the uterus incompletely palpable.
- Score 6: margins of the uterus cannot be delineated.

Measurements were taken on the pregnant uterine horn, with the probe positioned approximately 2 cm cranial to the bifurcation.

### 2.4. Uterine Content Sampling

Uterine secretions were collected on day 14 postpartum using a sterile plastic probe guided by a seeding rod protected by a covering layer. The probe was inserted up to the level of the uterine horns, and secretions were aspirated for microbiological analysis.

### 2.5. Uterine Biopsy

Uterine tissue samples were collected on day 14 postpartum using a 63 cm stainless steel biopsy rod with a 5 mm slot and a sharp edge. The rod was guided through the cervix, fixed on the uterine wall, and tissue samples were extracted using the sharp-edged slot. The samples were stored in 10% buffered formaldehyde for histopathological analysis. In the laboratory, the tissue samples were processed as follows: dehydration through a graded series of ethanol concentrations, clearing in xylene to prepare for embedding, embedding in paraffin wax, sectioning into thin slices of approximately 5 µm using a rotary microtome, and staining with hematoxylin and eosin (H&E) to evaluate general histological structures. Additional special stains were used when necessary to highlight specific features of interest, such as inflammatory cells or connective tissue changes.

The histological evaluation focused on assessing the integrity of the uterine epithelium, examining the uterine glands for structure and organization, identifying stromal edema by observing fluid accumulation in the stroma, and quantifying leukocyte infiltration, including neutrophils and macrophages, to evaluate the inflammatory response. Lower scores indicated healthier tissues, while higher scores reflected more significant pathological changes [9]. Statistical analysis of the scores was conducted to compare the two groups.

### 2.6. Clinical Monitoring and Reproductive Performance

All the animals were clinically examined throughout the study period to record the development of postpartum pathologies. Monitoring continued until the confirmation of a new pregnancy via ultrasonography or rectal palpation. Key reproductive performance parameters, including the calving interval and service period, were recorded and analyzed for both groups.

### 2.7. Economic Data Collection

Economic losses associated with retained placenta were estimated for both groups. These calculations included costs related to veterinary treatments, reduced milk yield, extended calving intervals, and potential culling and replacement expenses for non-recovering animals. The costs used for the economic calculations are found in Table 1.

### 2.8. Statistical Analysis

Data analysis was conducted using Microsoft Excel 2019. Continuous variables, such as uterine involution scores, calving interval, and service period, were tested for normality using Excel functions (e.g., mean, standard deviation, and histograms). For group comparisons, an independent *t*-test, Mann–Whitney U test, and chi-squared test methods were used.

Descriptive statistics (mean, median, and standard deviation) were calculated for all variables. *p*-values < 0.05 were considered statistically significant. This study design facilitated a comprehensive evaluation of the efficacy of the two treatment protocols in terms of uterine health, reproductive performance, and recovery in buffaloes with retained placenta.

The study protocol was reviewed and approved by the Ethics Commission of the University of Agricultural Sciences and Veterinary Medicine (USAMV) Cluj-Napoca, Romania, in accordance with the European Union animal welfare regulations and ethical guidelines for research involving animals.

## 3. Results

### 3.1. Uterine Involution

Uterine involution was assessed using the Grunert scoring method, with measurements taken on days 7, 14, 21, and 28 postpartum. Lower scores on this scale indicate better uterine retraction and smaller uterine horn size, representing normal physiological progression after calving. The results for group P (puerperal treatment) and group C (control) are summarized in Table 2.

Both groups exhibited a consistent decrease in uterine scores over the postpartum period, reflecting progressive uterine involution. On day 7, group P showed a slightly better involution score (4.7 ± 0.4) compared to group C (4.9 ± 0.5). This trend continued through day 28, with group P achieving a final mean score of 2.5 ± 0.3, compared to 3.0 ± 0.4 for group C. Although the differences between the groups were not statistically significant (*p* > 0.05), the results indicate a mild improvement in uterine recovery in the puerperal treatment group.

**Figure 1:**
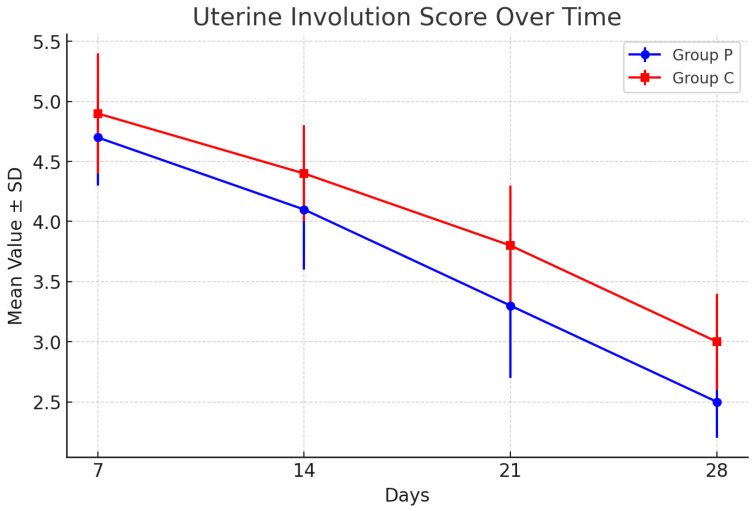
Uterine involution scores for groups P and C. Graphical representation of the mean values over time for groups P and C, with error bars representing the standard deviations.

The graphical representation of these results is shown in Figure 1, illustrating the decline in uterine involution scores over the 28-day observation period.

### 3.2. Microbial Flora

The analysis of microbial flora in uterine samples was conducted on day 14 postpartum. The microorganisms were classified into two categories: normal flora, consisting of commensal bacteria that maintain uterine health, and pathological flora, comprising microorganisms associated with uterine infections. The distribution of microbial flora between group P and group C is presented in Table 3.

Normal flora included beneficial microorganisms such as *Lactobacillus* spp. and *Bifidobacterium* spp., which are known to create a protective environment and prevent overgrowth of pathogenic bacteria. Pathological flora consisted of potential pathogens, including *Escherichia coli*, *Trueperella pyogenes*, and *Staphylococcus aureus*, which are frequently implicated in postpartum uterine infections and delayed recovery [10].

The results demonstrate a statistically significant (*p* < 0.05) difference between the groups in the composition of microbial flora. Group P exhibited a higher population of normal flora (85 ± 12 CFU/mL) compared to group C (67 ± 15 CFU/mL), suggesting that puerperal treatment fostered a uterine environment favorable to beneficial bacteria. Conversely, the presence of pathological flora was notably lower in group P (42 ± 10 CFU/mL) relative to group C (65 ± 12 CFU/mL), indicating better control of harmful microorganisms in the treatment group.

The most commonly isolated pathogenic microorganisms were *Escherichia coli*, *Trueperella pyogenes*, *Staphylococcus aureus*, and *Fusobacterium necrophorum*, all of which are frequently associated with postpartum uterine infections and endometritis. In the control group (group C), *E. coli* was the predominant pathogen, with an average concentration of 26 ± 7 CFU/mL, followed by *T. pyogenes* (18 ± 6 CFU/mL), *S. aureus* (11 ± 4 CFU/mL), and *F. necrophorum* (10 ± 3 CFU/mL). In contrast, the treatment group (group P) exhibited significantly lower concentrations of these pathogens, with *E. coli* at 15 ± 5 CFU/mL and *T. pyogenes* at 12 ± 4 CFU/mL (*p* < 0.05). The concentrations of *S. aureus* and *F. necrophorum* were also lower in group P (8 ± 3 CFU/mL and 7 ± 2 CFU/mL, respectively), though these differences were not statistically significant. Notably, the treatment group demonstrated a higher prevalence of beneficial microorganisms, particularly *Lactobacillus* spp. and *Bifidobacterium* spp., which play a crucial role in maintaining uterine health and preventing the overgrowth of pathogenic bacteria.

### 3.3. Histological Examinations

The uterine tissues of group P exhibited better preservation of epithelial integrity and a lower degree of stromal edema compared to group C. In group P, the epithelium appeared intact in most samples, with a well-organized glandular structure and minimal inflammatory infiltration. Conversely, tissues from group C demonstrated areas of epithelial desquamation, disorganized glandular morphology, and pronounced stromal edema. Leukocyte infiltration, a key indicator of inflammatory response, was notably higher in group C, with dense clusters of neutrophils and macrophages observed in the stroma and subepithelial layers.

The histological scoring system used in this study assigned higher values to more severe pathological changes. The scores for each parameter are summarized in Table 4, with lower scores indicating healthier uterine tissue. Statistical analysis showed significant differences (*p* < 0.05) between the two groups for all assessed parameters.

The epithelial integrity scores highlight the significant difference in tissue health between the groups. The lower scores in group P indicate minimal damage, whereas the higher scores in group C suggest notable epithelial disruption. Similar trends were observed in glandular morphology, with group P showing well-maintained glands compared to the disorganized appearance in group C. Stromal edema was markedly reduced in group P, demonstrating less fluid accumulation and better tissue recovery. Leukocyte infiltration was significantly lower in group P, indicating a reduced inflammatory response and faster resolution of postpartum uterine inflammation.

These findings suggest that the puerperal treatment in group P effectively supported uterine recovery, reducing histological markers of inflammation and tissue damage compared to the control group.

### 3.4. Reproductive Parameters

The reproductive performance of the buffaloes was assessed using three key parameters: service period, calving interval, and number of inseminations per pregnancy. Data were collected for both groups, group P and group C, and analyzed statistically to identify differences between the treatments. The results are summarized in Table 5.

The results revealed statistically significant differences (*p* < 0.05) in all three parameters, indicating that puerperal treatment contributed to improved reproductive performance in buffaloes.

Buffaloes in group P experienced a 24-day reduction in the service period, a 34-day shorter calving interval, and required 0.5 fewer inseminations per pregnancy compared to those in group C. These findings underscore the benefits of puerperal treatment in enhancing fertility and reducing reproductive inefficiencies.

### 3.5. Economical Implications

The economic evaluation revealed significant differences between the two treatment approaches regarding overall costs and cost per animal. For the 86 buffaloes included in the study, the classic treatment group incurred higher expenses compared to the puerperal treatment group due to increased insemination attempts, milk yield losses, and culling rates.

The treatment costs were €6.00 per animal for the classic treatment and €8.00 per animal for the puerperal treatment. This represented a minor difference of €2.00 per animal, favoring the classic treatment. However, the average number of artificial insemination attempts required to achieve pregnancy was €2.30 in the classic treatment group and €1.8 in the puerperal treatment group, resulting in an average cost difference of €16.00 per animal in favor of the latter.

Milk yield losses were more pronounced in the classic treatment group due to the longer service period, with an estimated additional 26 days of reduced milk production per buffalo. This resulted in a total cost of €1341 for the group, or €31.20 per animal. In contrast, the puerperal treatment group did not exhibit extended milk yield losses, significantly reducing costs.

Culling and replacement costs also varied considerably between the groups. The culling rate in the classic treatment group was 18%, resulting in eight animals being replaced, whereas the puerperal treatment group had a culling rate of 9%, with only four animals requiring replacement. This translated into costs of €7200 and €3600, respectively, highlighting the economic advantage of the puerperal treatment.

When all the parameters were combined, the average cost per buffalo was €250.60 in the classic treatment group compared to €127.70 in the puerperal treatment group. This equated to savings of €122.90 per animal in favor of the puerperal treatment. For the entire study, the total loss for the puerperal treatment group was €5491.10, while the classic treatment group incurred €10,877.80, underscoring the cost effectiveness of the puerperal approach (Table 6).

## 4. Discussion

Retained placenta in buffaloes represents a significant challenge in dairy and meat bovine production systems, as it is closely associated with postpartum complications, reduced reproductive performance, and economic losses. The failure of placental expulsion within 12 to 24 h after calving is influenced by multiple factors, including metabolic imbalances, infections, hormonal dysregulation, and inadequate uterine contractions [11,12]. The condition is frequently linked to dystocia, twin births, and nutritional deficiencies, leading to delayed uterine involution, increased susceptibility to bacterial infections, and subsequent reproductive disorders such as endometritis and infertility [13,14]. Given these detrimental effects, the development of effective therapeutic approaches is essential to enhance uterine recovery and restore reproductive efficiency.

The use of intrauterine therapies has been extensively explored as an alternative to conventional treatments involving manual placental removal and systemic antibiotic administration. The puerperal product used in this study contains a combination of vitamins, kaolin, boric acid, and neomycin, each contributing to uterine health and recovery. Vitamins, particularly A and E, play a crucial role in modulating immune responses and improving epithelial regeneration, thereby reducing the risk of uterine infections [14,15]. Kaolin, a natural adsorbent, aids in the removal of toxins and bacterial endotoxins, creating a favorable intrauterine environment [16]. Boric acid exhibits antimicrobial and antifungal properties, preventing secondary infections that can delay uterine involution [17]. The inclusion of neomycin and broad-spectrum antibiotics ensures bacterial load reduction, mitigating the progression of postpartum uterine infections [18].

A comparison of uterine involution between the two treatment groups revealed a faster regression of uterine dimensions in the Puerperal Treatment group compared to the Classic Treatment group. The uterine involution scores demonstrated a more rapid decline in the experimental group, suggesting enhanced uterine contractility and tissue remodeling. These findings align with previous studies that reported improved uterine recovery following intrauterine administration of antimicrobial and adsorbent agents [19,20]. In contrast, the Classic Treatment group exhibited prolonged uterine involution, which is consistent with literature indicating that manual placental removal may cause additional trauma and delay healing [21].

Microbiological analyses revealed distinct differences in uterine flora composition between the groups. The puerperal treatment group exhibited a lower bacterial load and a higher prevalence of normal commensal bacteria, suggesting that the treatment facilitated microbial balance within the uterus. Conversely, the classic treatment group had a higher incidence of pathogenic bacteria such as *Escherichia coli* and *Trueperella pyogenes*, which are strongly associated with metritis and endometritis [22]. These results corroborate previous research indicating that intrauterine antimicrobial treatments significantly reduce uterine infections and enhance reproductive outcomes [23,24].

Reproductive parameters, including the service period, the calving interval, and the number of inseminations required to achieve pregnancy, showed significant improvement in the puerperal treatment group. The reduction in the number of inseminations per conception and the shorter service period observed in this group are indicative of better uterine health and enhanced fertility. Previous studies have reported similar findings, where accelerated uterine involution and reduced bacterial contamination resulted in improved conception rates and reduced inter-calving intervals [25]. These improvements have significant implications for herd productivity and reproductive management, as prolonged service periods are associated with increased economic losses due to delayed milk production and extended calving intervals [26,27].

Histological examination further supported these findings, as the uterine tissues from the puerperal treatment group exhibited reduced inflammatory cell infiltration, increased epithelial integrity, and enhanced endometrial repair compared to the classic treatment group. The presence of chronic inflammatory changes in the latter group highlights the adverse effects of delayed uterine recovery on subsequent fertility. Studies by Chapwanya et al. (2009) [9] demonstrated that prolonged endometrial inflammation negatively impacts reproductive efficiency by altering the hormone signaling pathways necessary for successful implantation and pregnancy maintenance [28,29].

The economic implications of the two treatment approaches underscored the cost effectiveness of the puerperal treatment. Although the initial treatment cost was slightly higher, the reduction in artificial insemination attempts, milk yield losses, and culling rates significantly outweighed the additional expense. The estimated savings per buffalo in the puerperal treatment group compared to the classic treatment group highlighted the financial benefits of a more effective uterine recovery strategy. Similar economic assessments in dairy cattle have reported substantial cost reductions associated with improved postpartum management strategies [30,31,32].

Overall, the findings of this study indicate that puerperal treatment not only enhanced uterine recovery, but also improved reproductive performance and economic efficiency. By facilitating faster uterine involution, reducing bacterial contamination, and enhancing endometrial repair, this approach represents a promising alternative to conventional treatment methods. Further research involving larger sample sizes and long-term reproductive performance assessments would provide additional insights into optimizing postpartum management strategies in buffalo herds.

## 5. Conclusions

The study demonstrated that the intrauterine puerperal treatment containing vitamins, kaolin, boric acid, and neomycin significantly improved uterine recovery and reproductive performance in buffaloes with retained placenta compared to the classic treatment. Buffaloes treated with the puerperal product exhibited faster uterine involution, a lower prevalence of pathogenic bacterial flora, and improved reproductive parameters, including shorter service periods and calving intervals. Histological findings confirmed a more pronounced reduction in endometrial inflammation and better tissue regeneration, highlighting the formulation’s role in restoring reproductive health. From an economic standpoint, the improved fertility outcomes translated into reduced costs associated with additional inseminations, extended non-productive days, and milk yield losses, ultimately justifying the slightly higher treatment cost. The findings support the integration of intrauterine therapy with combined antimicrobial and supportive agents as a superior alternative to the conventional retained placenta management, contributing to both improved herd fertility and economic sustainability.

## Figures and Tables

**Table 1:** Estimated costs associated with retained placenta treatment.

Category	Cost (Euros)	Description
Classic treatment	6.00	Manual extraction and administration of 3 oxytetracycline pessaries
Puerperal treatment	8.00	Intrauterine infusion of a puerperal product containing vitamins, antibiotics, etc.
Culling and replacement costs	900.00 per culled animal	Cost of replacing a culled animal that failed to recover from retained placenta
Artificial insemination	20.00 per attempt	Cost per insemination, typically increased in cases with extended service periods
Milk yield loss	1.20 per day	Loss of income due to decreased milk production during delayed reproductive recovery

**Table 2:** Uterine involution scores for groups P and C (mean ± SD).

Group	Day 7	Day 14	Day 21	Day 28
P	4.7 ± 0.4	4.1 ± 0.5	3.3 ± 0.6	2.5 ± 0.3
C	4.9 ± 0.5	4.4 ± 0.4	3.8 ± 0.5	3.0 ± 0.4

P = group P (puerperal treatment), C = group C (control).

**Table 3:** Microbial flora composition in uterine samples (day 14 postpartum).

Group	Normal Flora (CFU/mL)	Pathological Flora (CFU/mL)
**P (mean value ± SEM)**	85 ± 12 ^a^	42 ± 10 ^c^
**C (mean value ± SEM)**	67 ± 15 ^b^	65 ± 12 ^b^

P = group P (puerperal treatment), C = group C (control), ^a,b,c^ statistically significant (*p* < 0.05) difference between the groups.

**Table 4:** Histological parameters of uterine tissue (day 14 postpartum).

Parameter	Group P (Mean ± SD)	Group C (Mean ± SD)	*p*-Value
Epithelial integrity	1.2 ± 0.3	2.8 ± 0.5	0.01
Glandular morphology	1.4 ± 0.4	3.1 ± 0.6	0.02
Stromal edema	1.5 ± 0.5	3.5 ± 0.7	0.01
Leukocyte infiltration	1.6 ± 0.4	4.2 ± 0.8	0.01

**Table 5:** Reproductive parameters of buffaloes in groups P and C.

Parameter	Group P (Mean ± SD)	Group C (Mean ± SD)	*p*-Value
Service period (days)	112 ± 15	136 ± 18	0.03
Calving interval (days)	398 ± 22	432 ± 25	0.04
Number of inseminations per pregnancy	1.8 ± 0.4	2.3 ± 0.5	0.036

**Table 6:** Economic costs per animal by treatment group.

Cost Component	Group C (€)	Group P (€)	Difference (€)
Treatment costs	6.00	8.00	+2.00
Artificial insemination	46.00	36.00	−10.00
Milk yield loss	31.20	0.00	−31.20
Culling and replacement	167.40	83.70	−83.70
Total cost per animal	250.60	127.70	−122.90

## Data Availability

Data are contained within the article.
